# Neural stem cell small extracellular vesicle-based delivery of 14-3-3t reduces apoptosis and neuroinflammation following traumatic spinal cord injury by enhancing autophagy by targeting Beclin-1

**DOI:** 10.18632/aging.102283

**Published:** 2019-09-28

**Authors:** Yuluo Rong, Wei Liu, Chengtang Lv, Jiaxing Wang, Yongjun Luo, Dongdong Jiang, Linwei Li, Zheng Zhou, Wei Zhou, Qingqing Li, Guoyong Yin, Lipeng Yu, Jin Fan, Weihua Cai

**Affiliations:** 1 Department of Orthopaedics, First Affiliated Hospital of Nanjing Medical University, Nanjing 210029, Jiangsu, China; 2 Department of Orthopaedics, Yancheng Third People’s Hospital, Yancheng 224000, Jiangsu, China

**Keywords:** NSC-sEVs, spinal cord injury, 14-3-3t, Beclin-1, autophagy

## Abstract

Neural stem cell-derived small extracellular vesicles (NSC-sEVs) play an important role in the repair of tissue damage. Our previous *in vitro* and *in vivo* studies found that preconditioning with NSC-sEVs promoted the recovery of functional behaviors following spinal cord injury by activating autophagy. However, the underlying mechanisms for such observations remain unclear. In this study, we further explored the mechanisms by which NSC-sEVs repair spinal cord injury via autophagy. We found that NSC-sEVs contain 14-3-3t protein, of which the overexpression or knockdown enhanced and decreased autophagy, respectively. In addition, 14-3-3t overexpression enhanced the anti-apoptotic and anti-inflammatory effects of NSC-sEVs, further promoting functional behavior recovery following spinal cord injury. The overexpression of 14-3-3t was used to further validate the *in vivo* results through a series of *in vitro* experiments. Conversely, knockdown of 14-3-3t attenuated the anti-apoptotic and anti-inflammatory effects of NSC-sEVs. Further studies also confirmed that NSC-sEVs increased Beclin-1 expression, with which 14-3-3t interacted and promoted its localization to autophagosome precursors. In this study, we found that NSC-sEVs deliver 14-3-3t, which interacts with Beclin-1 to activate autophagy. Our results indicate that 14-3-3t acts via a newly-discovered mechanism for the activation of autophagy by NSC-sEVs.

## INTRODUCTION

Spinal cord injury (SCI) includes primary and secondary injuries, which are characterized by varying degrees of damage to spinal nerve tissues. SCI affects patient behavior and function, causes severe paralysis, and can even result in death [1]. The pathophysiological mechanism of SCI is complex, in which apoptosis and an inflammatory response are the two major processes of secondary damage [2–4]. If the neurons are not effectively protected following SCI, activation of the neuronal apoptosis program can cause secondary and permanent neuronal damage, resulting in irreparable central nervous system damage [5]. SCI has serious consequences and is difficult to treat, and is one of the major issues that the modern medical community has not yet solved.

Stem cell transplantation may be a promising strategy to improve the recovery of motor, sensory and/or autonomic function following SCI [6]. Neural stem cells have the ability to self-renew and produce neurons, oligodendrocytes and astrocytes [7]. Studies have shown that neural stem cell transplantation has a unique neuroprotective function that promotes functional recovery after acute SCI [8, 9]. Previous studies have shown that NSCs improve motor function recovery after traumatic SCI via paracrine signalling [10].

NSC-sEVs not only contain cellular proteins and lipids, but also host cell mRNA and microRNA [11]. In addition, NSC-sEVs play a vital role in cell-to-cell communication by transporting proteins and RNA to target cells. Studies have shown that NSC-EVs are rich in specific miRNAs that mediate multiple functions under physiological and pathological conditions, including regulation of the adjacent microenvironment and promoting viral entry into cells. NSC-EVs can also behave as an independent functional metabolic unit, as a microglial morphogen, and regulate diverse aspects of brain function in adulthood, including the process of aging [12–15]. These results suggest that NSC-EVs play an important role in tissue damage and repair. Furthermore, in a recent study, we found that NSC-sEVs can repair SCI by activating autophagy [16]. This suggests that NSC-sEVs may also play an important role in the repair of SCI. However, the exact mechanism by which that may occur has not been thoroughly explored.

14-3-3 proteins are a class of highly conserved proteins that are widely present in different eukaryotic cells. 14-3-3 proteins have several isoforms, such as β, ε, η, γ, τ, σ, ζ, which have different expression levels in different tissues [17, 18]. The 14-3-3 family participates in various regulatory processes, including those involved in growth and development, signal transduction, checkpoint control, protein transport, cell cycle and apoptosis [19–21]. Furthermore, in the central nervous system (CNS), 14-3-3 proteins are usually induced in response to stresses, such as nerve damage and oxidation, possibly as a cell survival mechanism [22, 23]. Studies have shown that 14-3-3t protects cells from different types of stress, including ischemia, hypoxia, stress and viral attack [24–27]. Our previous studies also showed that increased 14-3-3 interaction with p-bad protected neurons from apoptosis [28].

Autophagy is an important defense and protection mechanism of the body. The body enhances autophagy to remove damaged and degraded proteins, as well as non-functioning organelles, for cellular recycling [29]. Autophagosome formation and maturation are complex processes that are highly regulated by multiple genes. Among such genes, Beclin-1 plays a key role in the production and maturation of autophagosomes. Autophagy is also critical for the protection of SCI, and the activation of autophagy plays a key role in reducing tissue damage. Multiple studies have shown that 14-3-3 proteins play important roles in the regulation of autophagy [20, 30, 31]. However, it is unclear whether the transmission of 14-3-3t by NSC-sEVs can activate autophagy to promote functional behavior recovery following traumatic SCI.

In this study, we discovered that the pre-treatment of neuronal cells with NSC-sEVs, which transferred 14-3-3t proteins, inhibited cells apoptosis and inflammation by activating autophagy. The results further confirmed that 14-3-3t binds to the autophagy-related protein, Beclin-1, to promote the formation of autophagosomes. These findings provide a theoretical basis for the future use of small extracellular vesicles as a new biological treatment for SCI.

## RESULTS

### NSC-sEVs induce autophagy activation *in vitro* and *in vivo*

High levels of extracellular glutamate can cause neuronal cell death (excitotoxicity), and glutamate-induced toxicity is one of the important pathogenic mechanisms of neuronal apoptosis and neurological dysfunction following SCI [32]. Therefore, to mimic neuronal cell damage after SCI *in vivo,* we used glutamate (Glu) to treat neurons *in vitro*. Since autophagy is critical for maintaining cell homeostasis and may contribute to anti-apoptosis and inflammatory effects, we examined the effect of NSC-sEVs on autophagy activation. As in the pretreatment method, nerve cells were treated with glutamate and cultured with or without NSC-sEVs. Autophagy-related indicators were then detected. Western blot results showed that compared with the Glu group and control, the autophagy-related protein, LC3BII, increased, while P62 decreased in the NSC-sEVs group (Supplementary Figure 1A–1C). The results of transmission electron microscopy showed that some autophagosomes appeared in neurons after Glu treatment. Compared with the Glu and control groups, the number of autophagosomes in NSC-sEVs-treated cells was higher (Supplementary Figure 1D, 1E). We then used confocal microscopy to examine autophagosomes in neuronal cells transiently transfected with mRFP-GFP-LC3. The results showed that the number of autophagosomes and autolysosomes in the NSC-sEVs group was higher (Supplementary Figure 1F, 1G).

To verify the *in vitro* results, western blots and immunofluorescence staining of P62 and LC3B were used to verify the effects of NSC-sEVs on autophagy *in vivo*. The western blot results showed that the expression of LC3BII was significantly higher in the NSC-sEVs-treated group than in the SCI group at 6 h and 24 h post-injury (Supplementary Figure 2A, 2B). Additionally, the expression of P62 was significantly lower than that in the SCI group, indicating that NSC-sEVs promoted autophagy after SCI, which was consistent with the *in vitro* results (Supplementary Figure 2A, 2B). Under fluorescence microscopy, P62/NeuN/DAPI double positives represented attenuated autophagy, while LC3/ NeuN/DAPI double positives represented autophagy in neuronal cells. The number of such positive neurons was then counted. The results indicated that compared with the SCI group, LC3-positive neurons in the NSC-sEVs group were significantly increased (Supplementary Figure 2E, 2F), while P62-positive neurons were significantly reduced (Supplementary Figure 2C, 2D), further confirming the *in vitro* results.

### NSC-sEVs transport 14-3-3t proteins, which are involved in autophagy activation

**Figure 1:**
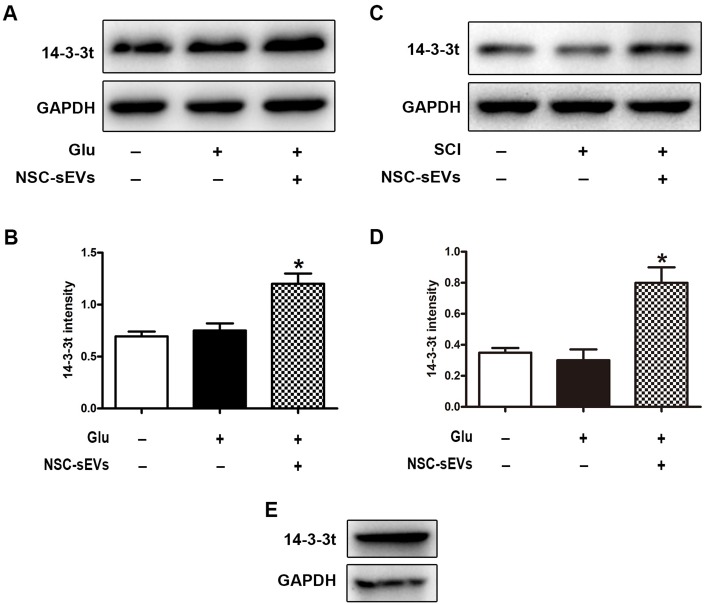
**NSC-sEVs transport 14-3-3t into neuronal cells and rat spinal cord tissue.** (**A**) Western blot was used to detect the expression of 14-3-3t in neuronal cells. (**B**) Semi-quantitative detection of 14-3-3t proteins expression level in neurons, normalized to GAPDH. (**C**) Western blot analysis of 14-3-3t expression in rat spinal cord tissue (**D**) Semi-quantitative detection of 14-3-3t proteins expression level in spinal cord tissues, normalized to GAPDH. (**E**) Western blotting was used to detect the expression of 14-3-3t and GAPDH in NSC-sEVs. * p < 0.05, compared to the Glu or SCI group. NSC-sEVs, neural stem cell-derived small extracellular vesicles; GAPDH, glyceraldehyde 3-phosphate dehydrogenase; Glu, glutamate; SCI, spinal cord injury.

The 14-3-3 family is a highly conserved protein family that interacts with more than 200 proteins and plays important biological functions in cells, including those in cellular signal transduction, apoptosis, and cell cycle regulation. Under normal growth conditions, 14-3-3 proteins can regulate the initiation of autophagy by interacting with the regulatory proteins, ULK1, Raptor, and TSC2, among others. The role of 14-3-3 protein in repairing SCI by NSC-sEVs is not well understood. Western blot analyses showed that NSC-sEVs could significantly promote the expression of 14-3-3t proteins in neuronal cells, compared to that of the Glu and control groups (Figure 1A, 1B). Thus, those data suggested that 14-3-3t proteins may be involved in the regulation of autophagy. At the same time, it was also found that 14-3-3t proteins in the NSC-sEVs group were significantly increased in spinal cord tissue, compared with expression in the Sham and the SCI groups (Figure 1C, 1D). Western blotting further indicated that NSC-sEVs encapsulated the 14-3-3t proteins (Figure 1E). Thus, those results suggested that NSC-sEVs can transmit the 14-3-3t proteins to target cells, and thereby exert certain biological functions.

### Overexpression or knockdown of 14-3-3t proteins in NSC-sEVs increases or decreases autophagy activity *in vitro,* respectively

**Figure 2:**
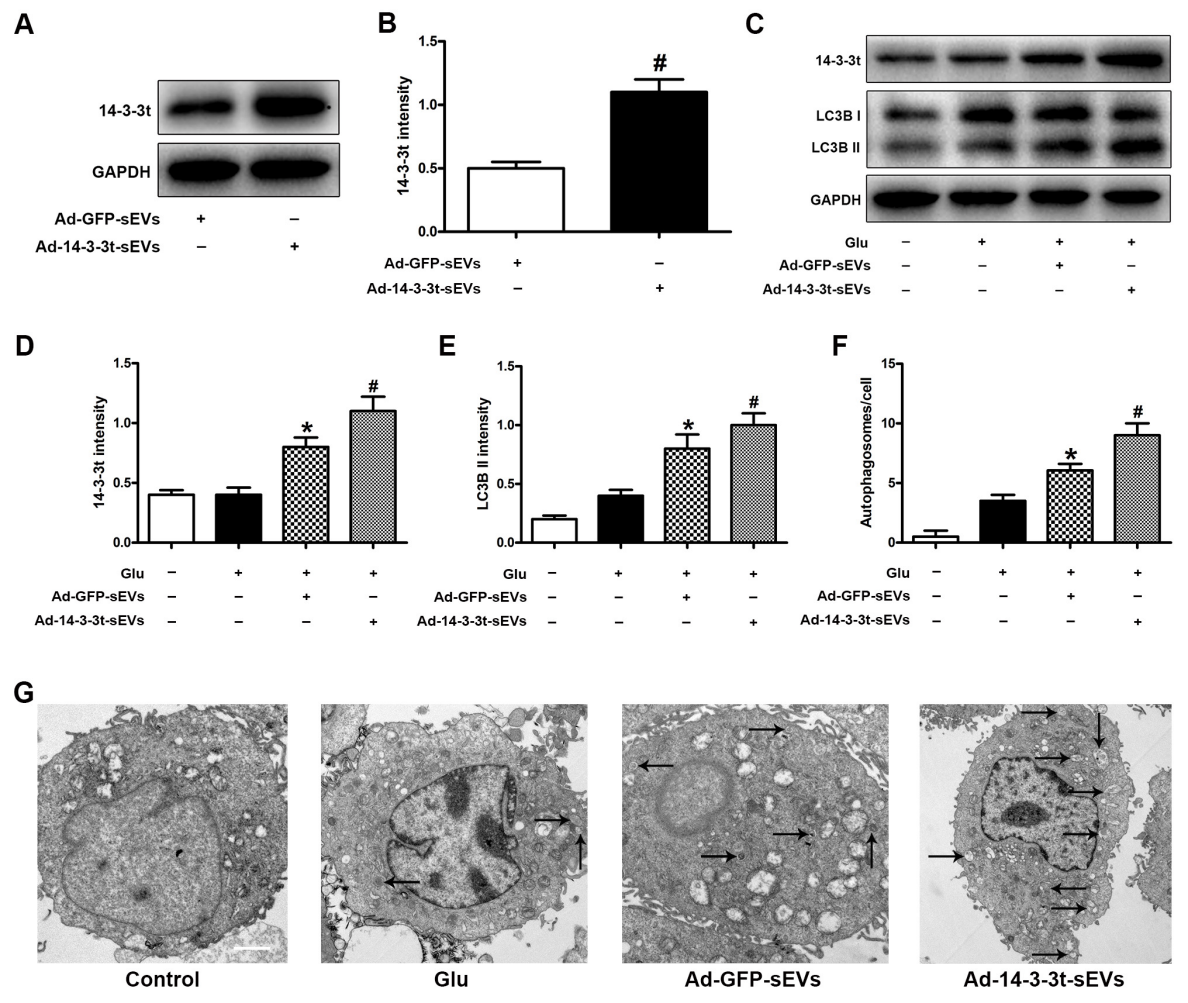
**Overexpression of 14-3-3t increased autophagic activity in neuron cells. Neuronal cells were treated with PBS, Ad-GFP-sEVs, and Ad-14-3-3t-sEVs for 24 h, followed by treatment with Glu for 24 h.** (**A**, **B**) Western blot assay for 14-3-3t expression in Ad-GFP-sEVs and Ad-14-3-3t-sEVs. (**C**) Western blot analyses of the expression of 14-3-3t and LC3B in neuronal cells. (**D**, **E**) Semi-quantitative detection of expression levels of 14-3-3t and LC3B, normalized to GAPDH. (**F**, **G**) Representative images of autophagosomes by TEM in neuronal cells. Scale bar = 2um. * p < 0.05, compared to the Glu group; # p < 0.05, compared to the Ad-GFP-sEVs group. GAPDH, glyceraldehyde 3-phosphate dehydrogenase; Glu, glutamate.

To determine the effect of 14-3-3t on autophagy, we overexpressed 14-3-3t in NSCs by adenovirus transfection (Ad-14-3-3t) and confirmed that the transduced proteins entered the NSC-sEVs using western blotting (Figure 2A, 2B). Western blots also showed an increase in 14-3-3t proteins in the Ad-14-3-3t-sEVs group, compared to that of the Ad-GFP-sEVs group (Figure 2C, 2D). Additionally, LC3BII was increased in the Ad-14-3-3t-sEVs group, compared to the Ad-GFP-sEVs group (Figure 2C, 2E). Electron microscopy revealed that the number of autophagosomes in neuronal cells treated with Ad-GFP-sEVs increased, compared to those observed in the Glu group (Figure 2F, 2G). In addition, the number of autophagosomes was higher in the Ad-14-3-3t-sEV group, compared to the Ad-GFP-sEVs group (Figure 2F, 2G).

To further elucidate the role of 14-3-3t in NSC-sEVs in autophagy, we knocked out 14-3-3t in NSCs by lentiviral transduction (sh14-3-3t) and confirmed the loss of the protein by western blotting. The expression of 14-3-3t proteins in NSC-sEVs were decreased in sh14-3-3t-sEVs, compared to shGFP-sEVs (Supplementary Figure 3A, 3B). The expression of 14-3-3t and LC3BII was also observed in neuronal cells by western blotting. The results showed that the expression of the two proteins in the sh14-3-3t-sEVs group was lower than that in the shGFP-sEVs group (Supplementary Figure 3C–3E). In addition, the number of autophagosomes in the sh14-3-3t-sEVs was reduced compared to that of the shGFP-sEVs group (Supplementary Figure 3F, 3G). This indicated that 14-3-3t overexpression in NSC-sEVs increased autophagy, while the loss of 14-3-3t attenuated autophagy.

### Overexpression of 14-3-3t enhances the repair of NSC-sEVs in functional behaviors after spinal cord injury

**Figure 3:**
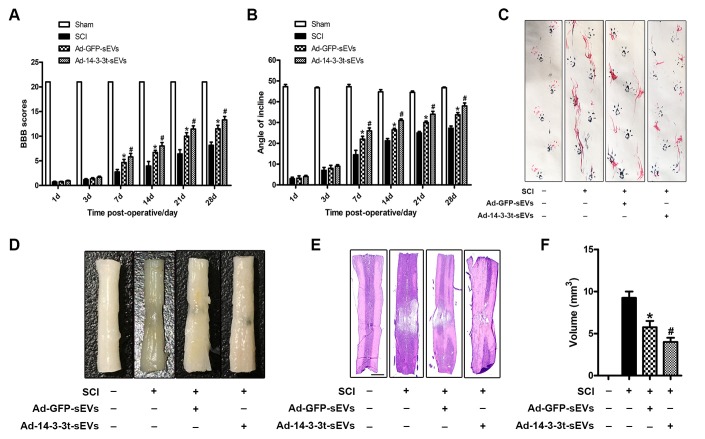
**Overexpression of 14-3-3t enhances the effect of NSC-sEVs on the repair of functional behaviors following spinal cord injury.** (**A**) BBB scores at different time points following spinal cord contusion. (**B**) Bevel test at different time points after spinal cord contusion. (**C**) Representative footprints of animal-walking 28 days after SCI. Blue: paw print on the front paw; red: paw print on the hind paw. (**D**) Gross morphology. (**E**) Representative H&E-stained sagittal section. Scale bar = 1000um. (**F**) Quantitative analysis of lesion volume between each group. * p < 0.05, compared to the SCI group; # p < 0.05, compared to the Ad-GFP-sEVs group. NSC-sEVs, neural stem cell-derived small extracellular vesicles; SCI, spinal cord; BBB, Basso, Beattie, and Bresnahan.

To verify the neuroprotective effect of 14-3-3t *in vivo*, we performed a footprint test and HE staining on SD rats following SCI to evaluate motor function and histological damage, respectively. The BBB score and bevel test were also used to evaluate the effect of NSC-sEVs on the recovery of motor function following SCI. Those results showed that motor function gradually improved 1 week after SCI injury. In the 2-4 weeks following SCI, the BBB scores of the Ad-GFP-sEVs and the Ad-14-3-3t-sEVs group continued to increase compared to the SCI group, with that of the Ad-14-3-3t-sEVs group being significantly increased (Figure 3A). The results of the bevel test also showed that the maximum dip angle of the Ad-14-3-3t-sEVs group was higher than that of the SCI group and the Ad-GFP-sEVs group (Figure 3B).

We also performed behavioral tests on rats 28 days after SCI. Specifically, the walking mode (gait) was evaluated by manually analyzing footprints. After SCI, the coordination of the forepaw-hind paw movements of all animals decreased significantly. Compared with the animals in the SCI group, the animals in the Ad-GFP-sEVs group and the Ad-14-3-3t-sEVs group showed significant recoveries of gait and motor coordination; however, the improvement in the Ad-14-3-3t-sEVs group was more pronounced (Figure 3C). The performance of the sham group rats remained unchanged throughout the test period.

HE staining was used to identify the traumatic lesion cavity due to cell loss, and to investigate whether 14-3-3t can significantly reduce the lesion volume. Four weeks after SCI, significant loss of SCI tissue was observed. However, compared to the SCI group, the Ad-GFP-sEVs group and the Ad-14-3-3t-sEVs group significantly reduced the area of tissue loss following trauma, with the reduction in the Ad-14-3-3t-sEVs group being more pronounced (Figure 3D–3F).

### Overexpression of 14-3-3t promotes the anti-apoptotic effect of NSC-sEVs in rats with spinal cord injury

**Figure 4:**
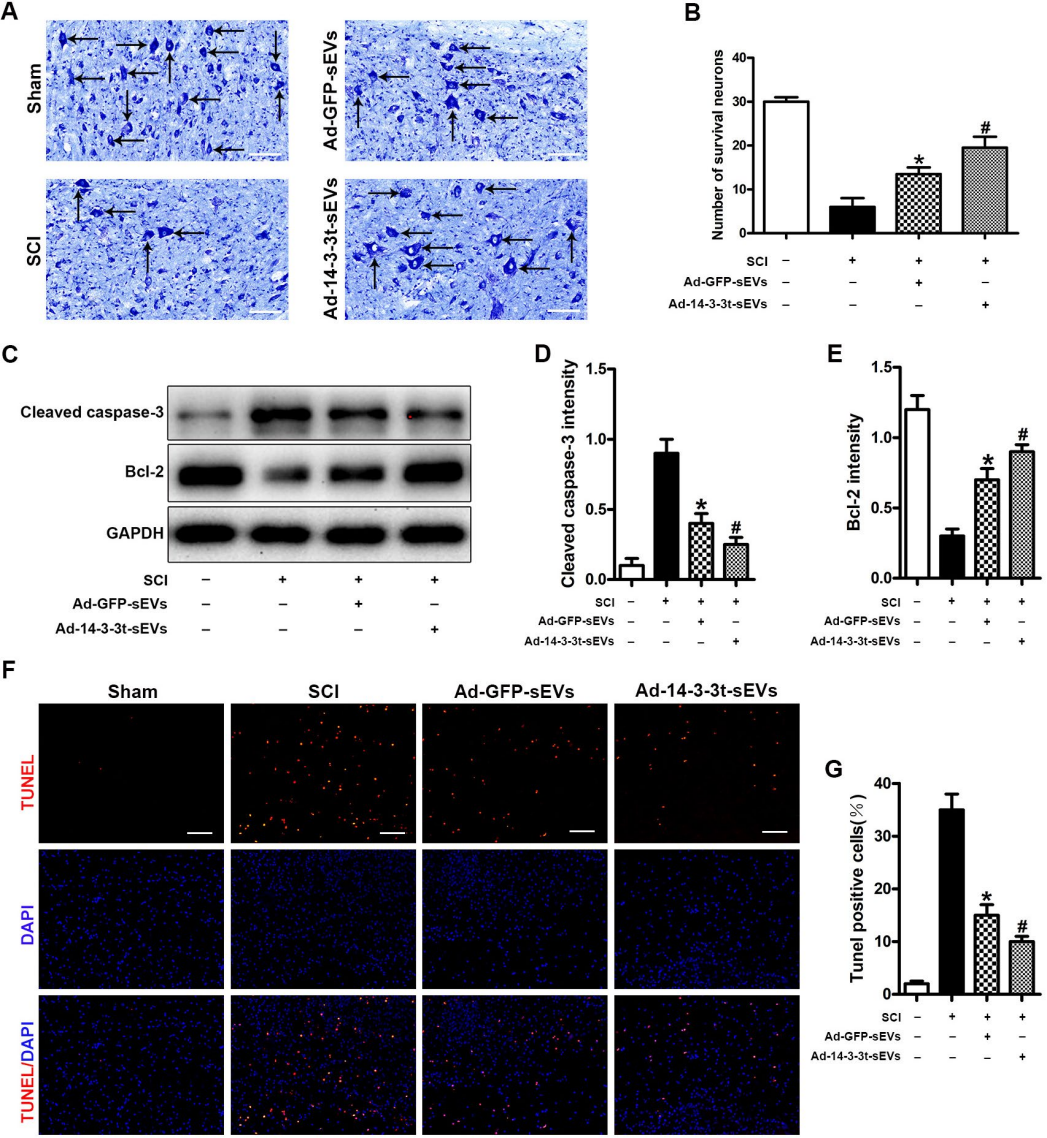
**Overexpression of 14-3-3t promotes the anti-apoptotic effect of NSC-sEVs in rats with spinal cord injury.** (**A**, **B**) Nissl staining indicating the number of motor neurons in each group. Scale bar = 50um. (**C**) Western blot analysis of apoptosis-related proteins after injury. (**D**, **E**) Semi-quantitative detection of expression levels of apoptosis-related proteins, normalized to GAPDH. (**F**) TUNEL staining was used to detect changes in neuronal apoptosis in each group. The proportion of TUNEL positive neurons in the NSC-sEVs group was lower than that in the SCI group. Scale bar = 100um. (**G**) Quantification of the number of TUNEL-positive neurons in each of the three experimental groups. * p < 0.05, compared to the SCI group; # p < 0.05, compared to the Ad-GFP-sEVs group. NSC-sEVs, neural stem cell-derived small extracellular vesicles; GAPDH, glyceraldehyde 3-phosphate dehydrogenase; TUNEL, terminal deoxynucleotidyl transferase-mediated dUTP nick end labeling assay; SCI, spinal cord.

The number of spinal motor neurons was measured by Nissl staining, and the degree of tissue damage after SCI was evaluated in the SCI model. Nissl staining showed that the number of neurons in the Ad-GFP-sEVs group was significantly higher than that in the SCI group 3 days after SCI (Figure 4A, 4B). The number of neurons in the Ad-14-3-3t-sEVs group was also higher than that in the SCI group, but was still lower than that in the sham group (Figure 4A, 4B). In addition, western blot analyses of spinal cord tissues showed that compared to the Ad-GFP-sEVs group, the Ad-14-3-3t-sEVs group had reduced expression of the apoptosis-related marker, cleaved caspase-3, and increased expression of the anti-apoptotic protein, bcl-2 (Figure 4C–4E).

The TUNEL method was used to further evaluate the apoptosis of neuronal cells in the SCI area. The number of TUNEL-positive cells after SCI was significantly increased compared to the Sham group, whereas treatment with Ad-14-3-3t-sEVs resulted in fewer TUNEL-positive cells (Figure 4F, 4G). Those findings indicated that the overexpression of 14-3-3t-sEVs promoted the anti-apoptotic effect of NSC-sEVs in rats with SCI.

### Overexpression of 14-3-3t enhances the anti-inflammatory effect of NSC-sEVs in rats with spinal cord injury

**Figure 5:**
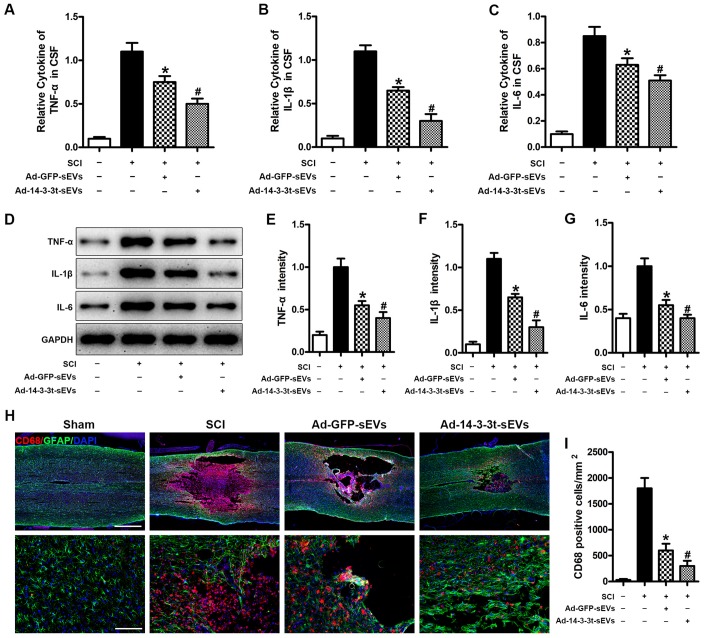
**Overexpression of 14-3-3t enhances the anti-inflammatory effect of NSC-sEVs in rats with spinal cord injury.** (**A**–**C**) ELISA was used to detect the expression levels of TNF-a, IL-1β and IL-6 in cerebral fluids. (**D**) Western blot detection of post-traumatic inflammation-related proteins. (**E**–**G**) Semi-quantitative detection of inflammation-related protein levels. (**H**) Representative immunohistochemical staining images of CD68 (red) and GFAP (green) on day 3 after spinal cord injury in the SCI and NSC-sEVs groups. All cell nuclei were stained with DAPI (blue). Scale bar = 1000 or 200um. (**I**) Analysis of the number of CD68+ microglia in the traumatic injury area. * p < 0.05, compared to the SCI group; # p < 0.05, compared to the Ad-GFP-sEVs group. NSC-sEVs, neural stem cell-derived small extracellular vesicles; DAPI, 4’,6-diamidino-2-phenylindole; GFAP, glial fibrillary acidic protein; SCI, spinal cord injury; TNF-a, tumor necrosis factor alpha; IL-1β, interleukin-1β; IL-6, interleukin-6.

To investigate the effect of 14-3-3t proteins on pro-inflammatory factors *in vivo*, we used ELISA to measure the levels of the cytokines, TNF-α, IL-1β and IL-6 in brain fluid following SCI. The results showed that TNF-α, IL-1β and IL-6 in the cerebrospinal fluid of the Ad-GFP-sEVs group were significantly decreased on the third day following SCI (Figure 5A–5C). The decrease was more significant in the Ad-14-3-3t-sEVs group (Figure 5A–5C). In addition, western blot analyses showed that the expression of TNF-α, IL-1β and IL-6 in the spinal cords of rats in the Ad-14-3-3t-sEVs group were also significantly lower than those in the Sham and SCI groups (Figure 5D–5G).

To further assess the effect of 14-3-3t on microglial activation after SCI, we quantified the number of CD68-positive (activated) microglia near lesions by immunostaining. Compared with the SCI group on the 3^rd^ day following SCI, the number of CD68-positive microglia in the Ad-GFP-sEVs group was significantly decreased, while the decrease in the Ad-14-3-3t-sEVs group was more significant (Figure 5H, 5I). Collectively, those data indicated that the overexpression of 14-3-3t enhanced the anti-inflammatory effects of NSC-sEVs in rats with SCI.

### Overexpression or knockout of 14-3-3t enhances or attenuates the anti-apoptotic effect of NSC-sEVs in neuronal cells, respectively

**Figure 6:**
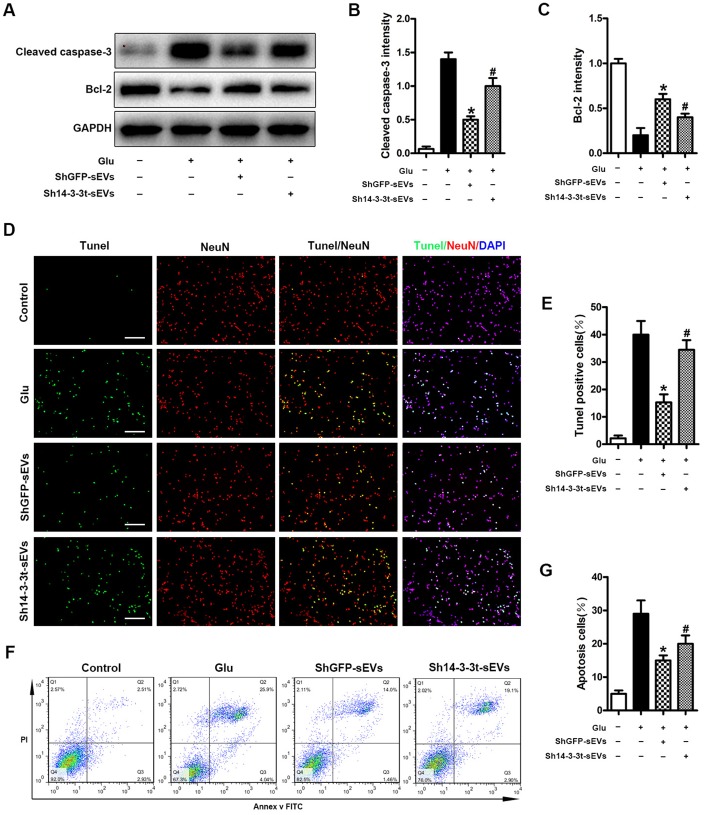
**Knockout of 14-3-3t attenuates the anti-apoptotic effect of NSC-sEVs in neuronal cells.** (**A**) Western blot analysis of changes in neuronal apoptosis-related proteins. (**B**, **C**) Semi-quantitative detection of relative expression levels of apoptosis-related proteins, normalized to GAPDH. (**D**) TUNEL detection of neuronal apoptosis. TUNEL-positive apoptotic cells (red). Nuclear staining using DAPI (blue). Scale bar = 100um. (**E**) Quantitative estimation of the proportion of apoptotic cells in each of the three experimental groups. (**F**) Annexin V/FITC/PI double staining and flow cytometry was used to detect neuronal apoptosis induced by Glu with or without NSC-sEVs. (**G**) Quantitative results of NSC-sEVs treatment and non-treatment of apoptotic neurons. * p < 0.05, compared to the Glu group, # p < 0.05, compared to shGFP-sEVs. NSC-sEVs, neural stem cell-derived small extracellular vesicles; DAPI, 4’,6-diamidino-2-phenylindole; FITC, fluorescein isothiocyanate; PI, propidium iodide; GAPDH, glyceraldehyde 3-phosphate dehydrogenase; Glu, Glutamate; TUNEL, terminal deoxynucleotidyl transferase-mediated dUTP nick end labeling assay.

Neuronal apoptosis caused by SCI can lead to neuronal cell death and neurological dysfunction. Therefore, we studied the effect of 14-3-3t proteins on apoptosis *in vitro*. The expression of Bcl-2 and Cleaved caspase-3 proteins was observed by western blotting in neuronal cells. Compared to the Glu and the sh14-3-3t-sEVs groups, the shGFP-sEVs group had significantly higher Bcl-2 levels and lower Cleaved caspase-3 levels (Figure 6A–6C). Moreover, compared to the shGFP-sEVs group, the number of TUNEL-positive neurons in the sh14-3-3t-sEVs group was significantly increased (Figure 6D, 6E). The changes in apoptosis were also evaluated using Annexin V-FITC/PI double staining flow cytometry after 14-3-3t knockout. Compared to the shGFP-sEVs group, sh14-3-3t-sEVs increased apoptosis (Figure 6F, 6G).

Following the overexpression of 14-3-3t, western blot analyses revealed that the level of Bcl-2 was significantly higher in the Ad-14-3-3t-sEVs group compared to the Glu and Ad-GFP-sEVs groups, while the expression of Cleaved caspase-3 was lower in the Ad-14-3-3t-sEVs group (Supplementary Figure 4A–4C). In addition, TUNEL staining revealed that the number of TUNEL-positive neurons in the Ad-14-3-3t-sEVs group was significantly reduced (Supplementary Figure 4D, 4E). Annexin V-FITC/PI double staining and flow cytometry further confirmed our results. The number of apoptotic cells in the Ad-14-3-3t-sEVs group was lower than that in the Glu and the Ad-GFP-sEVs groups (Supplementary Figure 4F, 4G). This suggested that 14-3-3t may further demonstrate our *in vivo* results by activating neuroautophagy in a Glu-induced excitotoxicity model.

### The effect of 14-3-3t overexpression or knockdown in NSC-sEVs on lipopolysaccharide-induced secretion of pro-inflammatory cytokines in microglia cells

**Figure 7:**
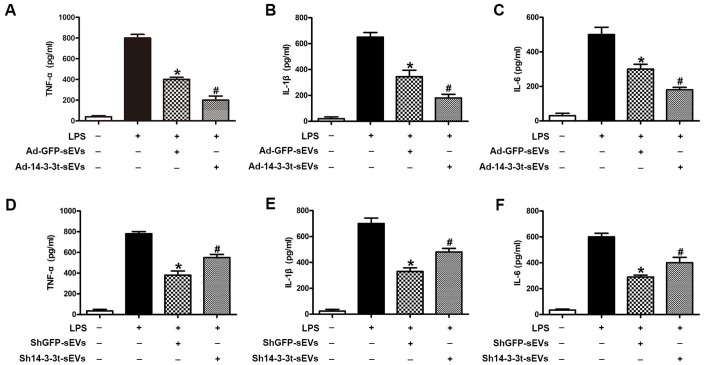
**The effect of 14-3-3t overexpression or knockdown in NSC-sEVs on lipopolysaccharide-induced secretion of pro-inflammatory cytokines in microglia cells.** (**A**–**C**) Following the overexpression of 14-3-3t in NSC-sEVs, ELISA was used to detect the release of the pro-inflammatory cytokines, TNF-a, IL-1β and IL-6 in microglia supernatants. (**D**–**F**) After 14-3-3t was knocked-out in NSC-sEVs, ELISA was used to detect the release of the pro-inflammatory cytokines, TNF-a, IL-1β and IL-6 in microglia supernatant. * p < 0.05, compared to the LPS group, # p < 0.05, compared to the Ad-GFP-sEVs or shGFP-sEVs groups. NSC-sEVs, neural stem cell-derived small extracellular vesicles; TNF-a, tumor necrosis factor alpha; IL-1β, interleukin-1β; IL-6, interleukin-6; LPS, lipopolysaccharide.

We investigated the effect of 14-3-3t proteins on lipopolysaccharide-induced inflammatory cytokine production in microglia. Compared to the PBS group, increased levels of proinflammatory factors (TNF-α, IL-1β, IL-6) in the supernatants of cells following treatment with LPS (5ng/mL) were detected by ELISA. When shGFP-sEVs and Ad-GFP-sEVs were pretreated with microglia, a significant decrease in the secretion level of inflammatory factors was observed (Figure 7A–7F). In addition, the secretion of pro-inflammatory factors in the Ad-14-3-3t-sEVs group was lower than that in the LPS and Ad-GFP-sEVs groups, but was still significantly higher than that in the PBS group (Figure 7A–7C). However, compared with the shGFP-sEVs group, the sh14-3-3t-sEVs group exhibited increased LPS-induced proinflammatory factor secretion (Figure 7D–7F). Those data suggest that 14-3-3t may affect the lipopolysaccharide-induced secretion of pro-inflammatory cytokines by activating autophagy, which is consistent with the *in vivo* results.

### 14-3-3t enhances autophagy by interacting with Beclin-1

**Figure 8:**
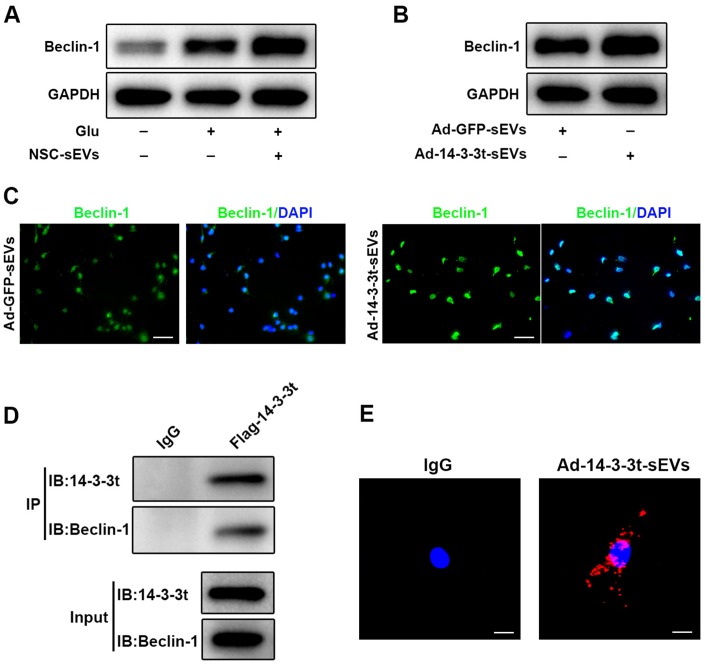
**14-3-3t enhanced autophagy by interacting with Beclin-1.** (**A**) Western blot assay for Beclin-1 in neuronal cells. (**B**) Western blot analysis of Beclin-1 in the Ad-GFP-sEVs and Ad-14-3-3t-sEVs groups in neuronal cells. (**C**) The expression of Beclin-1 proteins in neurons of the Ad-GFP-sEVs and Ad-14-3-3t-sEVs groups was detected by immunofluorescence. Scale bar = 50um. (**D**) Following the overexpression of flag-14-3-3t (from adenovirus) in neuronal cells, anti-Flag antibody was added. Co-immunoprecipitation results showed that 14-3-3t interacts with Beclin-1 in neuronal cells. (**E**) Indirect *in situ* PLAs were used to visualize protein interactions using red fluorophore-labeled oligonucleotides. Those assays showed that endogenous Beclin-1 interacted and co-localized with 14-3-3t in neurons following Glu treatment. Scale bar = 20um. Glu, Glutamate; PLA, Proximity ligation assay.

To further investigate the regulatory mechanism of 14-3-3t on autophagy, western blot analyses that pretreatment with NSC-sEVs increased the expression of Beclin-1 in neuronal cells, compared to the expression observed in the Glu and control groups (Figure 8A). Following the overexpression of 14-3-3t, western blot and immunofluorescence assays revealed that the expression of Beclin-1 was also increased, suggesting that 14-3-3t can promote the expression of Beclin-1 (Figure 8B, 8C). To further verify the relationship between 14-3-3t and Beclin-1, neuronal cells were transfected with Flag-14-3-3t overexpressing adenovirus, and the Flag antibody was added. The results of co-immunoprecipitation assays showed an interaction between 14-3-3t and Beclin-1, suggesting that 14-3-3t may promote autophagy through such interactions (Figure 8D).

Indirect *in situ* PLAs that visualize protein interactions using red fluorophore-labeled oligonucleotides showed that endogenous Beclin-1 interacted and co-localized with 14-3-3t in neurons following Glu treatment (Figure 8E). Taken together, these data indicate that NSC-sEVs transport 14-3-3t to neuronal cells, and that 14-3-3t increases Beclin-1 expression and interacts with it, facilitating its recruitment to autophagosome precursors, thereby induction of autophagy is increased.

## DISCUSSION

SCI is caused by direct or indirect damage to the spinal cord or related nerves, and can result in the incomplete or complete loss of sensation, strength, and other functions below the injury site [33]. After SCI occurs, a series of complex molecular and cellular events occur, including inflammatory responses, autophagy, and apoptosis [34–36]. In addition to primary injury, secondary tissue injury occurs following trauma caused by nerve cell death and is the main treatment disorder of SCI. Our previous studies have demonstrated that neural stem cell-derived small extracellular vesicles can promote the recovery of functional behavior in rats with SCI by activating autophagy. In this study, we further elucidated the mechanism by which neural stem cell-derived small extracellular vesicles repair SCI. Our studies showed that the overexpression of 14-3-3t in NSC-sEVs enhances the anti-apoptotic and anti-inflammatory effects of NSC-sEVs *in vitro* and *in vivo*. In contrast, 14-3-3t knockdown in NSC-sEVs *in vitro* attenuated such protection. Furthermore, this study indicated that 14-3-3t derived from NSC-sEVs activates autophagy by interacting with Beclin-1.

With the development of regenerative medicine and the ability of stem cells to differentiate into various cell types, stem cell transplantation is considered to have great prospects for the treatment of central nervous system diseases [37]. Studies have shown that neural stem cells have a unique neuroprotective function that promotes functional recovery after acute SCI [10, 38]. In fact, previous studies have shown that neural stem cell transplantations can reduce inflammation in the prototype of traumatic brain injury, hypoxic ischemic injury, SCI, and stroke [39, 40]. However, it is difficult to assess the optimal NSC purification and transplantation times. If NSCs are transplanted into the body at high densities, thrombosis will form [41]. The low survival rate of NSC *in vivo* is also a major problem that must be solved [41]. Therefore, although neural stem cell therapy has been successful in various animal disease models, due to the large physiological differences between humans and animals, many problems remain to be solved before being converted into clinical applications.

Small extracellular vesicles can be released into the extracellular environment by all cell types [42]. Small extracellular vesicles carry specific proteins, mRNA, long non-coding RNA and small non-coding RNA to adjacent cells [43]. Small extracellular vesicles have been found to play a role in tissue repair, immune response, organism development, and neuronal communication [44–48]. Our previous studies have shown that NSC-sEVs can repair SCI by promoting autophagy. However, few studies have focused on the function of proteins transported by NSC-sEVs in preventive medicine.

The role of autophagy as a pathway of degradation is critical in regenerative medicine. Many reports indicate that basal or physiological autophagy contributes to the maintenance of cell homeostasis and the quality control of proteins and subcellular organelles [49]. Pathological conditions or cellular stress can induce autophagy to survive as a cell's adaptive and protective mechanism [50]. Following the activation of autophagy, the expression of the autophagy-related protein, LC3BII, was up-regulated, while that of P62 expression was down-regulated. Studies have shown that in traumatic brain injury rat models, an increase in autophagy can reduce the damage to cells by treating the components of the lesion [51]. It is worth noting that autophagy induction as a self-protection mechanism has been confirmed in an experimental model of cell injury induced by traumatic SCI [52, 53]. In our previous study, we found that the repair of SCI by NSC-sEVs relied on autophagy. *In vitro*, we treated spinal cord neurons with a combination of the autophagy-specific inhibitor, 3MA, and found that the anti-apoptotic and anti-inflammatory effects of NSC-sEVs were reversed. However, the exact mechanism by which pretreatment with NSC-sEVs repairs SCI by activating autophagy is unclear. In this study, we demonstrate that NSC-sEVs-derived 14-3-3t proteins attenuate apoptosis and inflammatory responses following SCI by activating autophagy.

It is known that 14-3-3 proteins are involved in an increasing number of cellular biological processes, indicating the versatility of this ubiquitous family of eukaryotic adaptor proteins. In our study, we used LC-MS/MS analysis to identify that NSC-sEVs contain 14-3-3 proteins and the expression of 14-3-3t was very high. Furthermore, our study found that 14-3-3t proteins were up-regulated in the NSC-sEVs group, suggesting that NSC-sEVs can transport such proteins to neuronal cells. In addition, the overexpression of 14-3-3t enhances the anti-apoptotic and anti-inflammatory effects of NSC-sEVs *in vitro* and *in vivo*. In contrast, knockdown of 14-3-3t attenuated the anti-apoptotic and anti-inflammatory effects of NSC-sEVs in neurons and microglia. Those results indicate that 14-3-3t from NSC-sEVs plays a beneficial role in the repair of SCI.

In neurodegenerative diseases, different subtypes of 14-3-3 proteins have different regulatory functions on autophagy. 14-3-3ζ was reported to inhibit autophagy by interacting with the class III PI3K human vacuolar sorting protein 34 (hVps34) during nutrient deprivation [54]. Another study showed that 14-3-3ε exerts neuroprotective effects by reducing the formation of autophagosomes and attenuating I/R-induced neuronal apoptosis [55]. In contrast, 14-3-3ε siRNA enhances the formation of autophagosomes in neurodegenerative diseases. Many key regulatory proteins are involved in autophagy; 14-3-3 proteins play an additional role in regulating autophagy by binding to other proteins. Hao et al. showed that 14-3-3ζ prevented cisplatin-induced acute kidney injury by binding to ATG16L [56]. Weerasekara et al. showed that 14-3-3ζ promoted autophagy in hypoxia via interaction with phosphorylated Atg9 [57].

Beclin-1 plays an important role in autophagy. Under physiological and pathological conditions, the regulation of Beclin-1 may be an important mechanism for controlling autophagy [58]. Wang et al. found that 14-3-3t up-regulated the expression of Beclin-1 by E2F1 [31]. A significant reduction in Beclin-1 expression correlating with positive regulation of autophagy has been observed in U20S cell lines response expressing inducible or transient siRNA against 14-3-3t [30]. In this study, we demonstrated that overexpression of 14-3-3t enhanced autophagy activity. Furthermore, we found that 14-3-3t knockdown inhibited autophagy activity. This suggests that 14-3-3t is critical for the regulation of autophagy in the repair of SCI by NSC-sEVs in a rat model. This study also found that 14-3-3t activates autophagy by binding to Beclin-1 and promoting the localization of Beclin-1 to the outer surface of phagocytic cells.

In conclusion, this study revealed that the overexpression of 14-3-3t in NSC-sEVs promotes autophagy, which in turn enhances the anti-apoptotic and anti-inflammatory effects of NSC-sEVs *in vitro* and *in vivo*. In contrast, the knockdown of 14-3-3t in NSC-sEVs attenuated the anti-apoptotic and anti-inflammatory effects of NSC-sEVs *in vitro*. Furthermore, this study suggests that 14-3-3t derived from NSC-sEVs activates autophagy by interacting with Beclin-1. These findings provide a new method and theoretical basis for the treatment of SCI by extracellular vesicles.

## MATERIALS AND METHODS

### Isolation and identification of NSC-sEVs

Neural stem cells were obtained from 13.5 day-old fetal mice spinal cords and cultured in growth medium withwith 2% N2 (Gibco, Grand Island, NY, USA), 1% B27 (R&D Systems, Minneapolis, MN, USA), bFGF 20 ng/ml (R&D Systems), and EGF 20 ng/ml (R&D Systems). The growth medium was collected and centrifuged at 300 g for 10 mins, followed by centrifugation at 2000 g for 10 min at 4 °C. After centrifugation, the cell supernatant was sterilized by passing through a 0.22 μm filter to remove cell debris. The extracted NSC-sEVs were then diluted in PBS and stored at −80°C. The protein concentration of NSC-sEVs was quantified using a bicinchoninic acid assay (BCA; Thermo Fisher Scientific, Waltham, MA). The characterization of NSC-sEVs was performed by TEM, DLS and western blotting, as previously described [16].

### Primary spinal neuron culture

Primary neurons were extracted from embryonic (E16-E18) SD rats according to established protocols [59]. Briefly, cells were seeded on poly-D-lysine-coated plates (Corning Inc, Corning, NY, USA). For immunofluorescence staining, neurons were seeded in 24-well culture plates at 5×10^4^ cells/mL. For western blot assays, neurons were seeded at 1×10^6^ cells/mL in 6-well culture plates. After culture for 4 h, the seed plate medium was replaced with serum-free 96% neurobasal medium containing B27 (2%, w/v; Thermo Fisher Scientific), glutamine (0.5 mM; Thermo Fisher Scientific), penicillin (100 IU/mL), and streptomycin (100 mg/mL). Half of the medium was changed every 2 days and cell growth was observed under an inverted microscope. Cells were cultured for 7 days before use in experiments.

### Knockdown or overexpression of 14-3-3t in NSCs

In order to knockdown the expression of 14-3-3t proteins, a 14-3-3t shRNA was developed to silence the 14-3-3t genes. We combined the 14-3-3t shRNA oligonucleotides with the pGLVU6-puro vector to construct the Lenti-14-3-3t shRNA vectors. Lenti-shRNA was used as the negative control vector. The sequences of 14-3-3t shRNA were: forward, 5’-CACCGCTGGTTCAGAAGGCCAA ACTTTCAAGAGAAGTTTGGCCTTCTGAACCAGCTTTTTTG-3’, and reserve, 5’-GATCCAAAAAAGCTGG TTCAGAAGGCCAAACTTCTCTTGAAAGTTTGGCC TTCTGAACCAGC-3’. Lentiviruses (Lenti-14-3-3t shRNA or Lenti-shRNA) were used to transfect NSCs according to manufacturer's instructions. Western blotting was used to evaluate the efficiency of 14-3-3t knockdown.

The adenovirus expression vector (Ad-GFP) containing the 14-3-3t (Ad-14-3-3t) expression sequence and the empty adenovirus vector (Ad-GFP) were used as controls and transfected into NSCs according to the manufacturer's guidelines. The culture medium was changed after 24 h, and at 48 h post-transfection, cells were used for further analysis. The fractions were separated according to a previously-described method and designated as shGFP-sEVs, sh14-3-3t-sEVs, Ad-GFP-sEVs, and Ad-14-3-3t-sEVs.

### Double-labeled adenovirus mRFP-GFP-LC3 transfection and autophagy detection

Primary spinal cord neurons were prepared as described and were seeded on confocal dishes for 4 days, and then transfected with mRFP-GFP-LC3 lentivirus (Han Heng Biology, China), according to the manufacturer's protocol. Cells were divided into three groups: Control, Glu, and NSC-sEVs+Glu. Following treatment, cells were washed with PBS, fixed in 4% paraformaldehyde, and observed using laser confocal microscopy (Zeiss, Oberkochen, Germany, LSM 510). The number of yellow spots representing autophagic bodies and red spots representing autophagic lysosomes were enumerated.

### TEM assessment autophagy

After treatment, adherent neurons were detached with trypsin and centrifuged. The cell pellet was fixed with a pre-cooled 2% glutaraldehyde solution at 4 °C for 2 h, stained with 2% uranyl acetate solution for 2 h, and dehydrated in an acetone gradient of 50%, 70%, 90%, and 100%. The cells were then embedded and ultrathin sections were prepared for observation under an electron microscope (FEI Tecnai, Hillsboro, OR, USA).

### Cell apoptosis measurements by TUNEL staining or annexin V/FITC/PI double staining and flow cytometry, *in vitro*

Exposure to glutamate (Glu; 100 μM) was used as an *in vitro* model of SCI-induced cell death. Cultured primary spinal neurons with or without pre-treatment for 24 h with Ad-or sh14-3-3t-sEVs (100 μg/mL) were subjected to Glu treatment. Cells were then incubated with anti-NeuN (1:800, Abcam, USA) overnight at 4 °C after fixation, rupture, and blocking. The cells were washed with PBS and incubated with Alexa Fluor 488-conjugated goat anti-rabbit IgG antibodies (1:200, Jackson ImmunoResearch, USA) for 2 h, and then reacted with TUNEL staining solution (Roche, Basel, Switzerland) at 37 °C for 30 min in the dark, according to the manufacturer's instructions. The cells were counterstained for 5 min with DAPI (Beyotime Biotechnology, China) and observed by a fluorescence microscope (AXIO Vert.A1 and Imager A2; Carl Zeiss Microscopy GmbH, Jena, Germany). Apoptotic cells and total cells were counted in randomly-selected fields of view to calculate the proportion of TUNEL-positive (apoptotic) cells.

The apoptosis rate was also examined by flow cytometry. After the indicated treatment, cells were harvested by centrifugation at 1500 rpm for 5 min, and washed twice with PBS. The harvested cells were resuspended in FITC-labeled Annexin V (5uL; BD Biosciences) and PI (5uL; BD Biosciences) in darkness for 5 min, and washed three times with PBS. The cell apoptosis rate was then estimated by flow cytometry (FACSCalibur; BD Biosciences).

### Western blot analysis

Total protein was extracted from cells and tissues, and the protein concentration was measured using a BCA assay kit (as described above). Proteins were separated by sodium dodecyl sulfate-polyacrylamide gel electrophoresis (SDS-PAGE) and transferred onto polyvinylidene difluoride membranes. Membranes were blocked with 5% bovine serum albumin for 1 h at room temperature and incubated with antibodies against cleaved caspase-3 (1:1000, Cell Signal Technology, Danvers, MA, USA), LC3B (1:1000, Abcam, USA), Bcl-2 (1:1000, Abcam, USA), P62 (1:1000, Abcam, USA), 14-3-3t (1;1000, Santa Cruz, USA), Beclin-1 (1:1000, Abcam, USA), TNF-α(1:1000, Abcam, USA), IL-1β (1:1000, Abcam, USA), IL-6 (1:1000, Abcam, USA), and GAPDH (as a gel-loading control, 1:1000, Abcam, USA). Membranes were then incubated with HRP-conjugated secondary antibodies (1:2000, Thermo Fisher Scientific, USA) for 2 h at room temperature, followed by visualization of the immunolabeled bands using an enhanced chemiluminescence reagent (Thermo Fisher Scientific, USA). Protein expression levels were determined by densitometry using ImageJ software (NIH, Bethesda, MD).

### The expression levels of inflammatory cytokines were detected by ELISA

Primary microglia were harvested from postnatal day 3 rat pups, as described previously [60]. Microglia (2×10^5^ cells/mL) were plated on 48-well plates, pretreated with Ad-14-3-3t- or sh14-3-3t-sEVs (100 μg/mL) for 1 h or left untreated (control), and then stimulated with 5 ng/mL lipopolysaccharide (LPS; Sigma-Aldrich). After 24 h of stimulation, the cell supernatant was collected, and the levels of TNF-α, IL-1β, and IL-6 were detected by ELISA. The operation was conducted in strict accordance with the instructions of the ELISA kit.

### Co-immunoprecipitation (co-IP)

After treatment, cells were rinsed once with ice-cold PBS and lysed in 1 mL ice-cold buffer A (20 mM Tris-HCl, pH 7.4, 150 mM NaCl, 1% Triton X-100, 0.5% sodium deoxycholate, 12 mM glycerophosphate, 10 mM sodium fluoride, 5 mM EGTA, 2 mM sodium vanadate, 1 mM PMSF, 2 mg/mL aprotinin, and 2 mg/mL leupeptin) for 30 min. Lysates were centrifuged at 14,000 rpm for 15 min and the supernatants were collected. The supernatants were then immunoprecipitated with the indicated antibodies overnight at 4 °C using protein A/G magnetic beads. The beads were rinsed three times with buffer A and the eluent proteins were subjected to 12% SDS-PAGE and electrotransferred to nitrocellulose membranes for immunoblotting.

### Proximity ligation assay (PLA)

Cells were cultured in cover glass slide chambers (Thermo Scientific, 155360). After fixation with 4% PFA, cells were subjected to PLA using a Duolink detection kit and Detection Reagents Red (Sigma-Aldrich, DUO94004 [Detection Solution, DUO84004; Ligation Buffer, DUO82009; Amplification Buffer, DUO82050; Ligase; Polymerase]), according to the manufacturer's instructions with minor modifications. Briefly, permeabilized cells were blocked and incubated with primary antibodies overnight at 4 °C. After incubation with secondary antibodies conjugated to unique DNA probes (anti-mouse and anti-rabbit for two primary antibodies provided in the kit), a rolling circle amplification step was used to perform proximity ligation (<40nm) and circularization of the DNA. After the amplification process, replications of the DNA circle were labeled by complementary oligonucleotide probes and the signals were observed under a confocal microscope (LSM 510; Carl Zeiss). Representative cells from three fields of view were selected and photographed. All images were of single focal planes.

### Rat model of SCI and experimental groups

Healthy adult male Sprague-Dawley rats (body weight, 180-220 g) were purchased from the Animal Center of Nanjing Medical University (Nanjing, Jiangsu). The study was approved by the Ethics Committee of Nanjing Medical University. All procedures were in accordance with the National Institutes of Health Laboratory Animal Care and Use Guidelines.

The rat SCI model was established by the improved Allen method. As stated in previous studies, after injection of pentobarbital (50 mg/kg) in rats, laminectomy revealed the T10 spinal cord. The exposed back surface of the spinal cord was subjected to the weight-drop method using a 10 g rod (2.5 mm in diameter; C4p01-001; RWD Life Science Corp, Shenzhen, China) dropped from a height of 12.5 mm. After the injury, the spinal cord was irrigated with normal saline, the incision was sutured, and antibiotics were administered for three consecutive days. Manual bladder expression was performed 3 times per day until bladder function was restored. Cerebrospinal fluid was collected from rats according to our previous report [61].

Rats were randomly divided into four groups (n=6 per group), including the Sham group, SCI-only group, Ad-GFP-sEVs group, and the Ad-14-3-3t-sEVs group. The SCI group and the sEVs group were administered tail vein injections of physiological saline (PBS, 200 μL) or Ad-GFP- or Ad-14-3-3t-sEVs (200 μg of total protein of Ad-GFP- or Ad-14-3-3t-sEVs precipitated in 200 μL of PBS) immediately after SCI.

### Assessment of locomotor capacity

The exercise capacity assessment was based on the Basso-Beatie-Bresnahan exercise scale and the bevel test, consistent with previous reports. Exercise recovery was assessed on days 1, 3, 7, 14, 21 and 28 after SCI. The Basso-Beattie-Bresnahan (BBB) test has a score range of 0-21. The total score for severe neurological dysfunction is 0, while a score of 21 indicates normal performance. Assessments began at a fixed time in the morning of each testing day and were performed independently by two trained assessors who were blind to the treatment history. The bevel test was carried out on a test apparatus. The maximum angle at which the rat remained in its position for more than 5 seconds without falling was recorded. Behavioral assessments were performed at different time points by assessors blinded to the group information.

### Footprint analysis

Gait and motor coordination were assessed 28 days after surgery. The front and rear paws were coated with dyes of different colors. Rats were then placed on absorbent paper surrounded by a cage and encourage to walk in a straight line. The footprint pattern was then digitized and a representative image was used to assess coordination.

### Preparation of spinal cord slices

Rats were anesthetized with a lethal dose of pentobarbital. Cold salt water was then injected through the heart, followed by paraformaldehyde (PFA; 4% w/v) until the limbs and trunk became rigid. The lamina was then opened and the spinal cord was carefully cut, leaving the tissue intact. The tissue was fixed in PFA (4% w/v) for 24 h at 4 °C, and then transferred to PBS containing 20% and 30% sucrose (w/v). Tissue was then embedded in an optimal cutting temperature (OCT) compound, and sliced along the longitudinal axis at a thickness of 18 μm using a freezing cytotome. All sections were stored at -80°C until immunostaining.

### Hematoxylin and Eosin (H&E) Staining

To observe the size of the SCI area on day 28 post-surgery, H&E staining was performed according to the standard method. Briefly, slices were rinsed with distilled water and stained with alum hematoxylin. It was differentiated with 0.3% acid ethanol and stained with eosin Y. Samples were then dehydrated, cleaned, and packed. Images were collected using microscopes and the damaged areas were identified by severe tissue destruction or the loss of staining.

### TUNEL staining of spinal cord slices

Spinal cord sections were fixed, blocked, and incubated with the TUNEL reaction mixture (Roche, Basel, Switzerland) at 37 °C for 1 h. The nuclei were counterstained with DAPI. The proportion of TUNEL-positive neurons in each group of animals was enumerated under a fluorescence microscope.

### Immunofluorescence staining of spinal tissue

Spinal cord sections were permeabilized in Triton X-100/PBS solution (0.3% w/v) for 30 min, blocked using native goat serum-PBS solution (10%, v/v), and stained overnight at 4 °C with primary antibodies against the following proteins: CD68 (1:200, EMD Millipore Corp), GFAP (1:1000, Abcam, Cambridge, UK), P62 (1:1000, Abcam, USA), and LC3B (1:200, Abcam, USA), and NeuN (1:500, Abcam, USA). Sections were washed three times in PBS, and incubated with Cy3- or FITC-conjugated secondary antibodies (1:200, Jackson ImmunoResearch, USA) for 2 h at room temperature. Nuclei were then counterstained using DAPI, and fluorescent images were acquired. For each slide, the SCI lesion area was identified as the region lacking staining. All images were acquired using the same exposure time and conditions to facilitate comparisons between animals and groups.

### Nissl staining

The cytoplasmic Nissl substance in spinal cord sections was observed by cresyl violet staining on day 28 post-surgery. Briefly, sections were rinsed with distilled water and stained for 10 minutes in cresyl violet solution. After rinsing with distilled water, the sections were differentiated with 95% ethanol, washed with xylene, and fixed with neutral balsam. Apoptotic neurons shrank or contained vacuoles. Normal neurons had relatively larger, more complete cells, and large round nuclei. Five regions were randomly selected and observed by an inverted microscope using a researcher blinded to the experimental group.

### Statistical analysis

Data and images were processed and analyzed using IBM SPSS Statistics v17.0 software. Data are expressed as the mean ± standard deviation of at least three independent experiments. To analyze the changes in BBB score, the angle of incline over time, and the differences between groups, we used a two-factor repeated measures analysis of variance (RT-ANOVA) with a Bonferroni's post hoc correction for multiple comparisons. All tests were two-tailed and a p-value < 0.05 was accepted as statistically significant for all tests.

## Supplementary Material

Supplementary Figures
