# Supplementary material for: Neural stem cell small extracellular vesicle-based delivery of 14-3-3t reduces apoptosis and neuroinflammation following traumatic spinal cord injury by enhancing autophagy by targeting Beclin-1
Source: Aging (Albany NY). 2019 Sep 28;11(18):7723–45. doi: 10.18632/aging.102283 (PMC6782003; doi:10.18632/aging.102283)
Supplement: Supplementary Figures [file aging-11-102283-s001.pdf]

SUPPLEMENTARY FIGURES

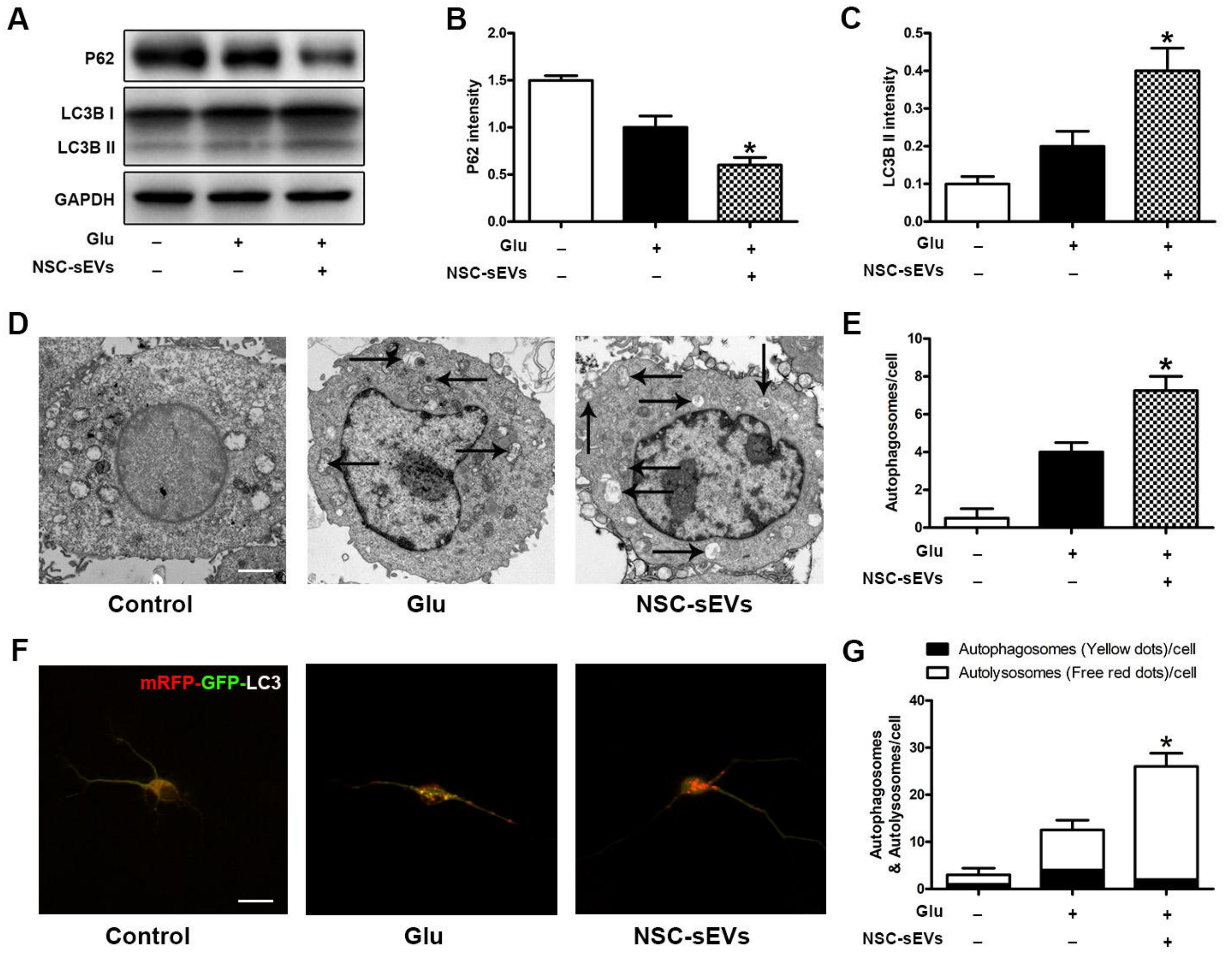

Supplementary Figure 1. NSC-sEVs induce autophagy in neurons *in vitro*. (**A**) Western blot detection of the neuronal autophagy markers, LC3B and P62. (**B**, **C**) Semi-quantitative detection of autophagy-related protein expression levels. (**D**, **E**) Transmission electron microscopy of neuronal autophagosomes. Representative images show typical autophagosomes in NSC-sEVs-treated cells. Scale bar = 2um. (**F**, **G**) Laser confocal microscopy of the autophagic flux of mRFP-GFP-LC3 transfected neuronal cells. Autophagosomes are labeled with red and green fluorescence (yellow spots); autophagic lysosomes are labeled with red fluorescence (red spots). The NSC-sEVs group had a larger number of yellow and red spots compared to the Glu group. Scale bar = 20um. * $p < 0.05$, compared to the Glu group. NSC-sEVs, neural stem cell-derived small extracellular vesicles; Glu, glutamate.

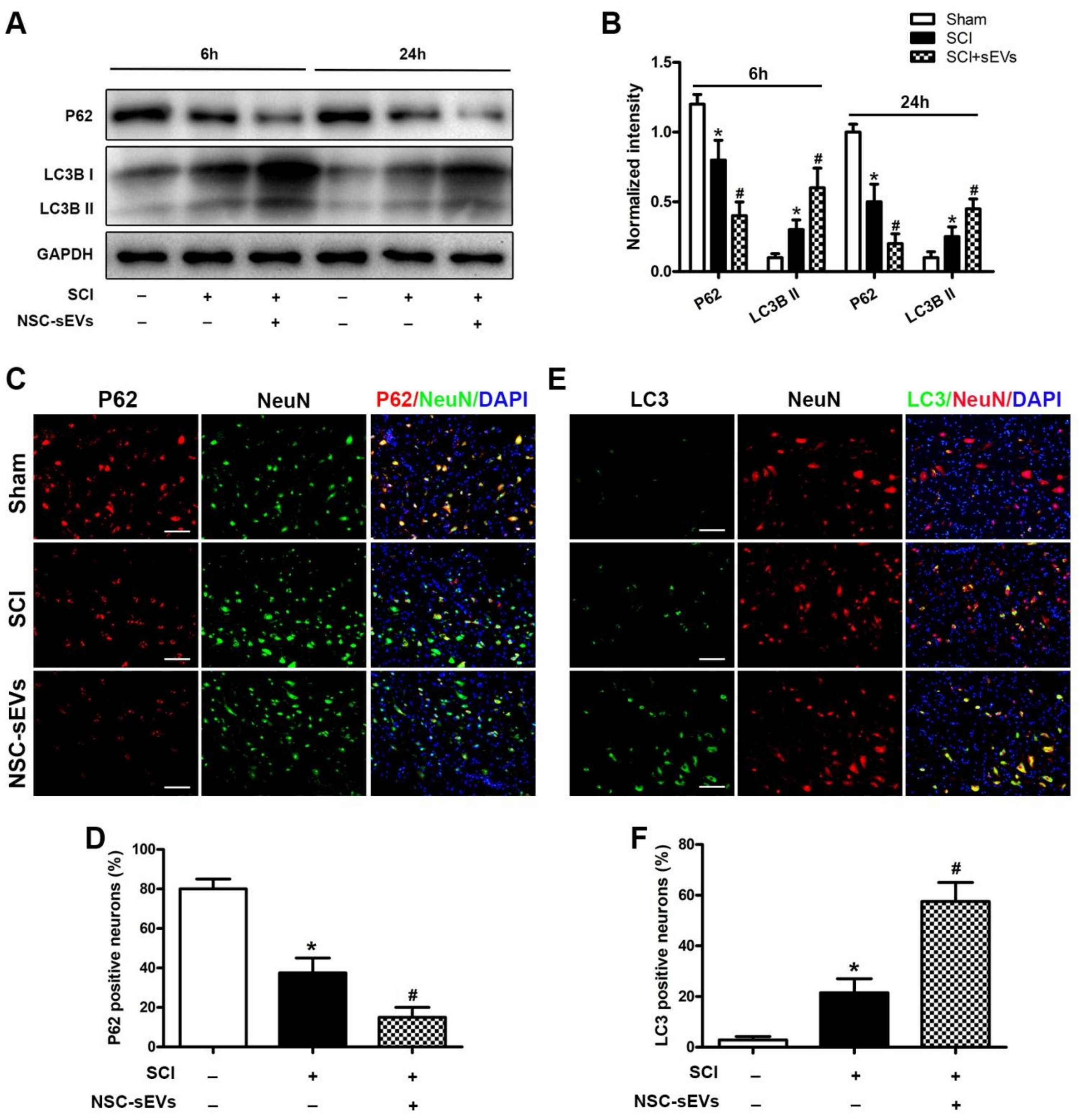

Supplementary Figure 2. NSC-sEVs promotes autophagy following SCI in rats. (**A**) Western blot analysis of autophagy-related proteins at 6 h and 24 h after injury. (**B**) Semi-quantitative detection of autophagy-related protein expression levels, normalized to GAPDH. (**C**, **D**) The number of P62/NeuN/DAPI double positive neurons was observed under a fluorescence microscope. Compared with the SCI group, P62-positive neurons in the NSC-sEVs group were significantly decreased. Scale bar = 20um. (**E**, **F**) The number of LC3/NeuN/DAPI double positive neurons was observed under a fluorescence microscope. Compared with the SCI group, LC3-positive neurons in the NSC-sEVs group were significantly increased. Scale bar = 20um. * $p < 0.05$, compared to the Sham group; # $p < 0.05$, compared to the SCI group. NSC-sEVs, neural stem cell-derived small extracellular vesicles; SCI, spinal cord injury; GAPDH, glyceraldehyde 3-phosphate dehydrogenase.

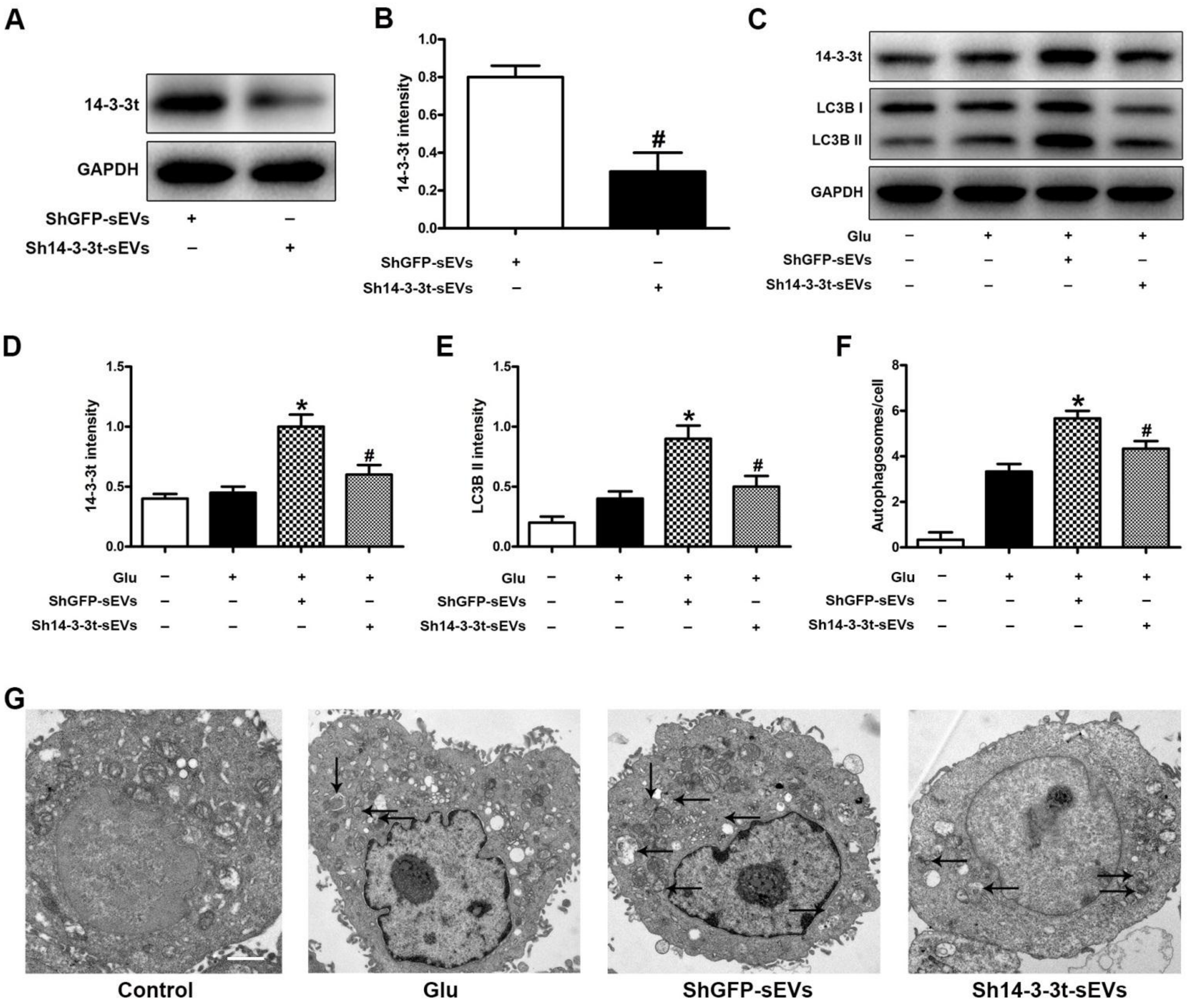

Supplementary Figure 3. Knockdown of 14-3-3t alleviated autophagic activity in neuronal cells. Neuronal cells were treated with PBS, shGFP-sEVs, and sh14-3-3t-sEVs for 24 h, and were then treated with Glu for 24 h. (**A**, **B**) Western blot assay for 14-3-3t expression in shGFP-sEVs and sh14-3-3t-sEVs. (**C**) Western blot analyses of the expression of 14-3-3t and LC3B in neuronal cells. (**D**, **E**) Semi-quantitative expression of expression levels of 14-3-3t and LC3B, normalized to GAPDH. (**F**, **G**) Representative images of autophagosomes by TEM in neuronal cells. Scale bar = 20um. * $p < 0.05$, compared to the Glu group; # $p < 0.05$, compared to the shGFP-sEVs group. GAPDH, glyceraldehyde 3-phosphate dehydrogenase; Glu, glutamate.

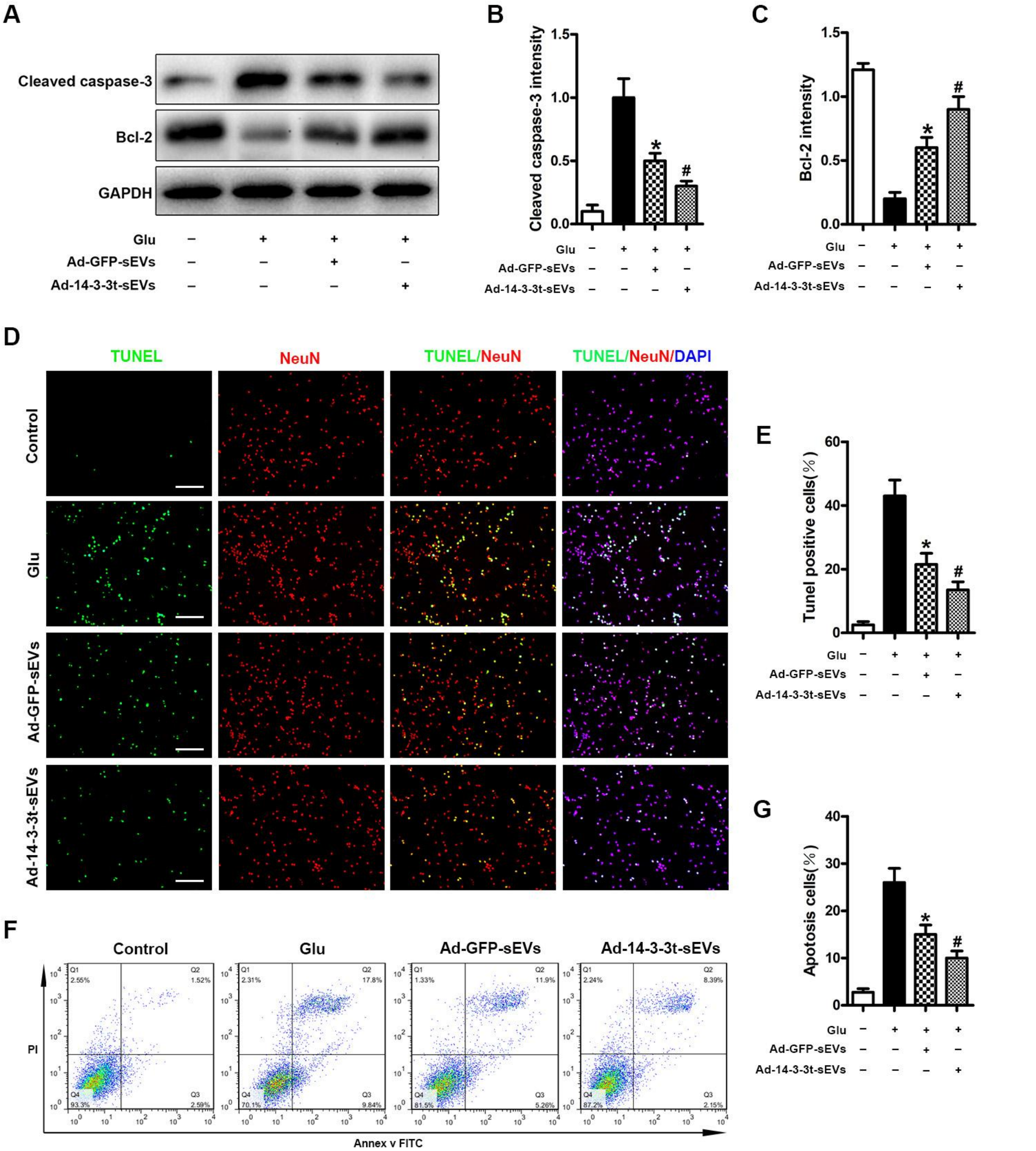

Supplementary Figure 4. Overexpression of 14-3-3t enhances the anti-apoptotic effect of NSC-sEVs in neuronal cells. (**A**) Western blot analysis of changes in neuronal apoptosis-related proteins. (**B**, **C**) Semi-quantitative detection of relative expression levels of apoptosis-related proteins, normalized to GAPDH. (**D**) TUNEL detection of neuronal apoptosis. TUNEL-positive apoptotic cells (red). Nuclear staining using DAPI (blue). Scale bar = 100um. (**E**) Quantitative estimation of the proportion of apoptotic cells in each of the three experimental groups. (**F**) Annexin V/FITC/PI double staining and flow cytometry was used to detect neuronal apoptosis induced by Glu with or without NSC-sEVs. (**G**) Quantitative results of NSC-sEVs treatment and non-treatment of apoptotic neurons. * $p < 0.05$, compared to the Glu group, # $p < 0.05$, compared to the Ad-GFP-sEVs group. NSC-sEVs, neural stem cell-derived small extracellular vesicles; DAPI, 4’,6-diamidino-2-phenylindole; FITC, fluorescein isothiocyanate; PI, propidium iodide; GAPDH, glyceraldehyde 3-phosphate dehydrogenase; Glu, Glutamate; TUNEL, terminal deoxynucleotidyl transferase-mediated dUTP nick end labeling assay.
